# Photoluminescent Hydroxylapatite: Eu^3+^ Doping Effect on Biological Behaviour

**DOI:** 10.3390/nano9091187

**Published:** 2019-08-22

**Authors:** Ecaterina Andronescu, Daniela Predoi, Ionela Andreea Neacsu, Andrei Viorel Paduraru, Adina Magdalena Musuc, Roxana Trusca, Ovidiu Oprea, Eugenia Tanasa, Otilia Ruxandra Vasile, Adrian Ionut Nicoara, Adrian Vasile Surdu, Florin Iordache, Alexandra Catalina Birca, Simona Liliana Iconaru, Bogdan Stefan Vasile

**Affiliations:** 1 Faculty of Applied Chemistry and Materials Science, Department of Science and Engineering of Oxide Materials and Nanomaterials, University Politehnica of Bucharest, 060042 Bucharest, Romania; 2 National Centre for Micro and Nanomaterials, University Politehnica of Bucharest, 060042 Bucharest, Romania; 3 National Research Center for Food Safety, University Politehnica of Bucharest, 060042 Bucharest, Romania; 4 Multifunctional Materials and Structures Laboratory, National Institute of Materials Physics, 077125 Magurele, Romania; 5 Ilie Murgulescu Institute of Physical Chemistry, 060021 Bucharest, Romania; 6 Faculty of Veterinary Medicine, Department of Biochemistry, University of Agronomic Science and Veterinary Medicine, 011464 Bucharest, Romania

**Keywords:** europium doped hydroxylapatite, photoluminescence, MTT assay, oxidative stress assessment, fluorescent microscopy

## Abstract

Luminescent europium-doped hydroxylapatite (Eu_X_HAp) nanomaterials were successfully obtained by co-precipitation method at low temperature. The morphological, structural and optical properties were investigated by scanning electron microscopy (SEM), transmission electron microscopy (TEM), *X*-ray diffraction (XRD), Fourier Transform Infrared (FT-IR), UV-Vis and photoluminescence (PL) spectroscopy. The cytotoxicity and biocompatibility of Eu_X_HAp were also evaluated using MTT (3-(4,5-dimethylthiazol-2-yl)-2,5-diphenyltetrazolium bromide)) assay, oxidative stress assessment and fluorescent microscopy. The results reveal that the Eu^3+^ has successfully doped the hexagonal lattice of hydroxylapatite. By enhancing the optical features, these Eu_X_HAp materials demonstrated superior efficiency to become fluorescent labelling materials for bioimaging applications.

## 1. Introduction

Bioceramics can be defined as the category of ceramics used in repairing and replacing processes of damaged and diseased parts of skeletal system [1,2]. Their biocompatibility varies from inert ceramic oxides to bioresorbable materials. One of the most used bioresorbable ceramics for biomedical applications are calcium orthophosphates (CaP’s) [3,4]. The CaP’s give hardness and stability to the tissues and can be found in teeth, bones, and tendons. Starting with Ca/P molar ratio equal to 0.5 and finishing with 2.0, there are 11 known non-ion substituted calcium orthophosphates, among which the most used one is hydroxylapatite (HAp) [5,6].

HAp synthesis, with its diverse morphologies, structures and textures, has attracted much interest in academic and industrial research for many heterogeneous catalysis applications [7,8,9]. Numerous synthetic routes for obtaining hydroxylapatite were developed over time, and can be divided in four main categories: (1) wet methods [10,11,12], (2) dry methods [13], (3) microwave-assisted methods [14,15,16], ball-milling [17,18,19] or ultrasound methods [20,21], and (4) miscellaneous methods [22]. Depending on the reagents and conditions, each category offers several variations [23,24].

Stoichiometric hydroxylapatite (Ca_10_(PO_4_)_6_(OH)_2_) is the most similar material to the mineral component of human hard tissues and therefore it is considered the ideal substance for bone defects restorations [25,26,27]. Keeping the same geometry while accepting a big variety of anions and cations is one of the most important structural characteristics of hydroxylapatite [28,29]. Synthetic HAp can also be doped with several metal ions in order to improve its properties, like bioactivity, degradation rate, antibacterial characteristics, luminescence and magnetic properties [30,31,32,33,34].

A photoluminescent material is the most promising candidate for clinical applications and implantation. The biocompatibility is not the only important feature, a longer lifetime of luminescence being also an significant benefit in practical applications [35,36].

Photoluminescence is a very important and useful mechanism in in situ investigations for tissue engineering, surgery, tissue restoration, etc. Using of organic fluorescent molecules for labelling it was a popular practice in clinical trials for many years. Recent studies use inorganic components, even in form of nanoparticles, to replace photoluminescent organic compounds. Because of the toxicity and the nano-size of this particles, the usage of these materials represents a challenge yet [37,38].

Due to high values of the Stokes shift and long lifetime of the excited state, lanthanide coordination compounds are the perfect materials for bioimaging. Europium complexes possess this unique luminescent properties and beside this Europium combine high photoluminescence quantum yields (PLQYs) with the emission in the long wavelength range, which can easily penetrate through the tissues [39,40,41].

The luminescence of the europium (III) and terbium (III) complexes is especially sensitive to changes in the structure and coordination environment of ions and depends substantially on the interaction with the analyte. The intensive luminescence and characteristic Stark structure of Eu^3+^ luminescence spectra allow registering fine changes in the structure of the coordination sphere of a rare-earth ion upon surrounding impact. Luminescent lanthanide-containing complex compounds can be applied as optical chemosensors in detection of anions, cations, gases, etc. [42].

The new generation of biomaterials with multifunctional europium (III)-doped HAp scaffolds has shown remarkable development. The luminescent multifunctional biomaterials show potential for use in various biomedical applications such as smart drug delivery, bioimaging, and photothermal therapy. In this generation, the Eu^3+^ ion has been widely used as traceable fluorescence probe due to well-known dopant narrow emission spectral lines by visible-light excitation caused by shielding by the 5 s and 5 p orbitals. The spectral shapes depend on the local ion symmetry and forbidden f–f transitions. Thus, Eu^3+^ is a sensitive optical probe for the dopant site environment because of its characteristic luminescence properties [43].

## 2. Experimental

### 2.1. Materials

All the reagents for synthesis, including calcium nitrate tetrahydrate (Ca(NO_3_)_2_·4H_2_O, 99.0%, Sigma-Aldrich, St. Louis, MI, USA), europium-(III) nitrate hexahydrate (Eu(NO_3_)_3_·6H_2_O, 99.9%, Alfa Aesar, Haverhill, MA, USA), ammonium phosphate dibasic (NH_4_)_2_HPO_4_, 99.0%, Alfa Aesar, Haverhill, MA, USA), sodium hydroxide (NaOH, 25% solution, Alfa Aesar, Haverhill, MA, USA) were used as received, without further purification. Deionised water was used for the experiment.

### 2.2. Synthesis

The biocompatible photoluminescent europium-doped hydroxylapatite (Eu_X_HAp) nanomaterials have been synthesized by co-precipitation method. In order to obtain Eu_X_HAp powders, appropriate amounts of calcium nitrate tetrahydrate and europium-(III) nitrate hexahydrate were dissolved in deionized water, under vigorous stirring at room temperature, thus obtaining solution A. Meanwhile, a solution B was prepared by dissolving an appropriate amount of ammonium phosphate dibasic in deionized water, under vigorous stirring at room temperature. The atomic ratio Ca/P and [Ca^+^Eu]/P was 1.67, while the atomic ratio Eu/(Eu^+^Ca) was varied between 0 and 50%. Solution B was added dropwise to solution A, at 80 °C, under vigorous stirring, while adjusting and maintaining the pH of the resulting suspension at 10, by adding NH_4_OH (25%) solution, for 2 h. Pure HAp was synthesized following the same methodology, except the Eu^3+^ precursor addition. The resulting suspensions were matured for 24 h. The precipitate was then filtered and washed several times with deionized water, until the pH values were close to 7. Finally, the resulting precipitates were dried at 80 °C for 50 h in an air oven.

### 2.3. Samples Notation

Eu_X_HAp represents Eu–doped hydroxylapatite, where X is equal to the used europium content (X_Eu_ = 0.05, 0.1, 0.15, 0.2, 1.0, 5.0). The correspondent Eu-free hydroxylapatite is noted HAp (X_Eu_ = 0).

### 2.4. Morphological and Structural Characterization

X-ray diffraction (XRD) studies were carried out using a PANalytical Empyrean diffractometer at room temperature, with a characteristic Cu X-ray tube (λ Cu K_α1_ = 1.541874 Ǻ) with in-line focusing, programmable divergent slit on the incident side and a programmable anti-scatter slit mounted on the PIXcel3D detector on the diffracted side. The samples were scanned in a Bragg - Brentano geometry with a scan step increment of 0.02° and a counting time of 255 s/step. The XRD patterns were recorded in the 2θ angle range of 20°–80°. Lattice parameters were refined by the Rietveld method, using the HighScore Plus 3.0 e software. The morphology of the samples was analyzed using a Quanta Inspect F50 FEG (field emission gun) scanning electron microscope with 1.2 nm resolution, equipped with an energy-dispersive X-ray (EDX) analyzer (resolution of 133 eV at MnK_α_, Thermo Fisher, Waltham, MA, USA) on sample covered with a thin gold layer. The high-resolution TEM images of the samples were obtained on finely powdered samples using a Tecnai G^2^ F30 S-Twin high-resolution transmission electron microscope from Thermo Fisher (former FEI) (Waltham, MA, USA).The microscope operated in transmission mode at 300 kV acceleration voltage with a TEM resolution of 1.0 Å. FTIR spectra were recorded with a Nicolet iS50R spectrometer (Thermo Fisher Waltham, MA, USA), at room temperature, in the measurement range 4000–400 cm^−1^. Spectral collection was carried out in ATR mode at 4 cm^−1^ resolution. For each spectrum, 32 scans were co-added and converted to absorbance using OmincPicta software (Thermo Scientific, Waltham, MA, USA). Raman spectra were recorded at room temperature on a Horiba Jobin-Yvon LabRam HR spectrometer equipped with nitrogen cooled detector. The near–infrared (NIR) line of a 785 nm laser was employed for excitation and the spectral range went from 500 to 1200 cm^−1^. UV-Vis diffused reflectance spectra were obtained using an Able Jasco V-560 spectrophotometer (PW de Meern, Netherlands,) with a scan speed of 200 nm/s, between 200 and 850 nm. The fluorescence spectra were measured by using a Perkin Elmer LS 55 fluorescence spectrophotometer (Arkon, OH, USA). Spectra were recorded with a scan speed of 200 nm/s between 350 and 800 nm, and with excitation and emission slits widths of 7 and 5 nm, respectively. An excitation wavelength of 320 nm was used.

### 2.5. Cellular Viability Assays

#### 2.5.1. Quantitative In-Vitro Evaluation of Biocompatibility—MTT Assay

MTT assay is a quantitative colorimetric method, which allows evaluation of cell viability and proliferation, and cytotoxicity of different compounds. The method is based on reduction of MTT tetrazolium salt (3-(4,5-dimethylthiazolyl)-2,5-diphenyltetrazolium bromide) to dark blue formazan. Reduction by mitochondrial enzymes (especially succinate dehydrogenase) is an indication of cell/mitochondrial integrity. Formazan, insoluble in water, can be solubilized with isopropanol, dimethylsulfoxide or other organic solvent. The optical density (DO) of solubilized formazan is evaluated spectrophotometrically, resulting in a color-absorbent-color-counting function of the number of metabolic active cells in the culture.

The human mesenchymal amniotic fluid stem cells (AFSC) were used to evaluate the biocompatibility of Eu_X_HAp nanoparticles. The cells were cultured in DMEM medium (Sigma-Aldrich, Saint Luis, MI, USA) supplemented with 10% fetal bovine serum, 1% penicillin and 1% streptomycin antibiotics (Sigma-Aldrich, Saint Luis, MI, USA). To maintain optimal culture conditions, medium was changed twice a week. The biocompatibility was assessed using MTT assay (Vybrant® MTT Cell Proliferation Assay Kit, Thermo Fischer Scientific, Waltham, MA, USA). Briefly, the AFSC were grown in 96-well plates, with a seeding density of 3000 cells/well in the presence of Eu_X_HAp for 72 h. Then 15 mL Solution I (12 mM MTT) was added and incubated at 37 °C for 4 h. Solution II (1 mg Sodium Dodecyl Sulphate + 10 ml HCl, 0,01M) was added and pipettes vigorously to solubilize formazan crystals. After 1 h the absorbance was read using spectrophotometer at 570 nm (TECAN Infinite M200, Männedorf, Switzerland).

#### 2.5.2. Oxidative Stress Assessment—GSH-Glo Glutathione Assay

The GSH-Glo Assay is a luminescent-based assay for the detection and quantification of glutathione (GSH) in cells or in various biological samples. A change in GSH levels is important in the assessment of toxicological responses and is an indicator of oxidative stress, potentially leading to apoptosis or cell death. The assay is based on the conversion of a luciferin derivative into luciferin in the presence of GSH. The reaction is catalyzed by a glutathione S-transferase (GST) enzyme supplied in the kit. The luciferin formed is detected in a coupled reaction using Ultra-Glo Recombinant Luciferase that generates a glow type luminescence that is proportional to the amount of glutathione present in cells. The assay provides a simple, fast and sensitive alternative to colorimetric and fluorescent methods and can be adapted easily to high-throughput applications.

AFSC were seeded at a density of 3000 cells in 300 µL of Dulbecco’s Modified Eagle’s medium (DMEM) supplemented with 10% fetal bovine serum and 1% antibiotics (penicillin, streptomycin/neomycin) in 96-well plates. Twenty-four hours after seeding, cells are treated with Eu_X_HAp and incubated for 72 h.

The working protocol consisted of adding 100 µL 1X GSH-Glo Reagent and incubating at 37 °C for 30 min. Then, 100 µL Luciferin DeectionReagent was added and incubated at 37 °C for an additional 15 min. At the end of the time, the wells were well homogenized and then the plate was read on the luminometer (MicroplateLuminometerCentro LB 960, Berthold, Germany).

#### 2.5.3. Qualitative In-Vitro Evaluation of Biocompatibility—Fluorescent Microscopy

The biocompatibility of the Eu_X_HAp was also evaluated by fluorescent microscopy, using RED CMTPX fluorophore (Thermo Fischer Scientific, Waltham, MA, USA), a cell tracker for long-term tracing of living cells. The CMTPX tracker was added in cell culture treated with Eu_X_HAp nanomaterials and the viability and morphology of the AFSC was evaluated after 5 days. The CMTPX fluorophore was added in the culture medium at a final concentration of 5 µM, incubated for 30 min in order to allow the dye penetration into the cells. Next, the AFSC were washed with PBS and visualized by fluorescent microscopy. The photomicrographs were taken with Olympus CKX 41 digital camera driven by CellSense Entry software (Olympus, Tokyo, Japan).

## 3. Results and Discussions

### 3.1. X-ray Diffraction

**Figure 1:**
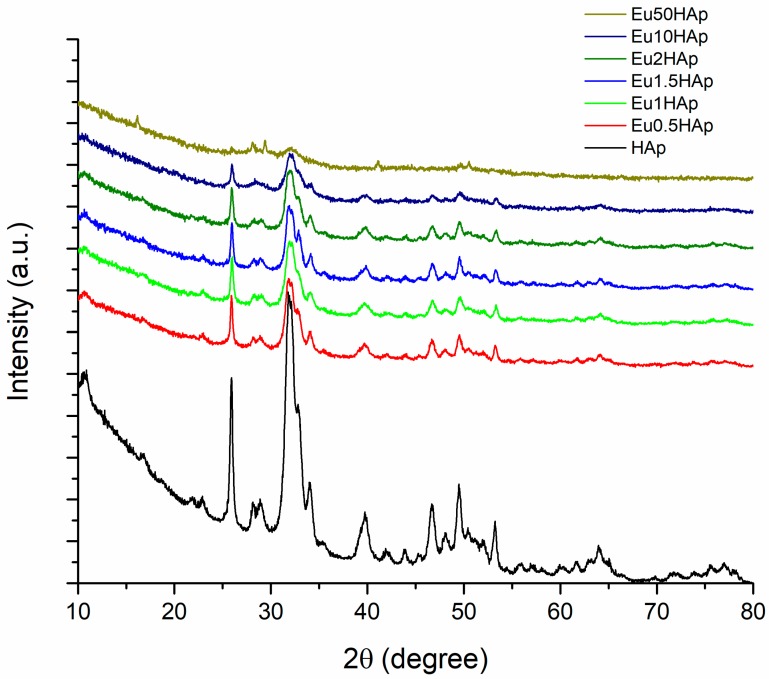
*X*-ray diffraction patterns of hydroxylapatite (HAp) and Eu_X_HAp.

The *X*-ray diffraction patterns of Eu-doped hydroxylapatite, with different concentration of europium and with pure HAp are shown in Figure 1.

**Figure 2:**
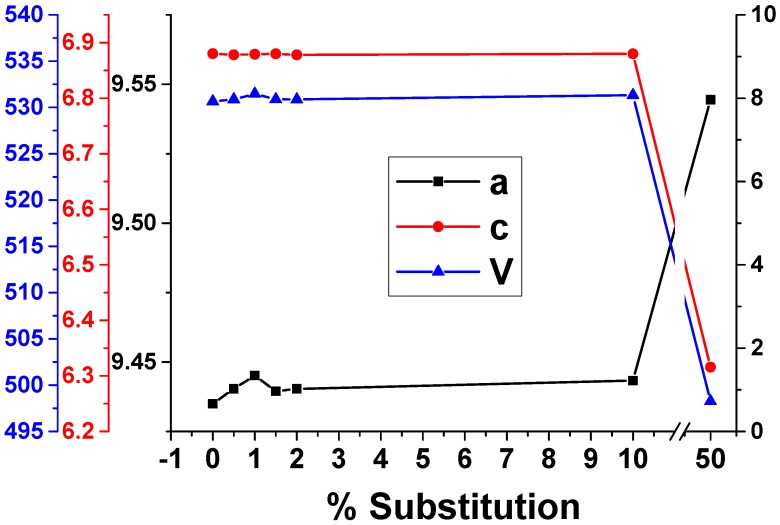
Unit cell parameters versus substitution degree for HAp and Eu_X_HAp.

Pristine HAp (Figure 1) is shown as a single phase calcium phosphate material (ICDD PDF4+ no.04-021-1904 [44]), with a crystallinity of 33.9% and an average crystallite size of 10.84 nm (Table 1). The XRD patterns of all Eu_X_HAp samples indicate only the pure crystalline hexagonal HAp phase (according to ICDD PDF4+ no.04-021-1904 [44] of the space group P6_3_/m, in consistence with literature [45] up to a substitution degree of 10%. For 50% substitution, Eu(OH)_3_ secondary phase in a mass ratio of 27.6% has occurred (according to ICDD PDF4+ no. 01-083-2305 [46]). This means that for 50% substitution, the limit of solubility of Eu in HAp lattice was exceeded. The intensities of *X*-ray peaks decrease when the Eu-doping level increases up to 2%, indicating an interference of Eu^3+^ with HAp crystal structure. Also, the peak position is influenced by Eu for Ca substitution as they shift to higher angle values which suggest decreasing of unit cell parameters. Table 2 and Figure 2 illustrate the values of unit cell parameters *a*, *c* and *V* and the agreement indices of the Rietveld analysis (*R_exp_*, *R_p_*, *R_wp_* and χ^2^), which indicate the quality of the fit.

Hydroxylapatite crystallizes in a hexagonal symmetry with lattice parameters *a* = *b* ≠ *c* and space group *P6_3_/m*. In this structure, PO_4_ tetrahedral form basic structural units, while the coordination around the distinct Ca sites defines the Ca1O1_3_O2_3_ metaprism and the distorted Ca2O1O2O3_4_(OH) polyhedron. Thus, the formula per unit cell may be expressed as Ca1_4_Ca2_6_(PO1O2O3_2_)_6_(O_H_H)_2_ [47]. Taking into consideration that the ionic radius of Eu^3+^ (0.947 Å) is smaller than that of Ca^2+^ (1 Å) and that small ions are preferentially substituted at Ca1, in this case Eu^3+^ most probably substitutes Ca^2+^ from site 2 [48,49]. While a low concentration does not induce significant changes (Figure 2), when looking at higher substitution degree (10% and 50%) it may be concluded that there is an increase of *a*-axis and a decrease of *c*-axis parameters which is consistent with the results obtained for Al^3+^ substitution by Fahami et al. [49]. Moreover, the decrease of unit cell volume proves the incorporation of Eu^3+^ in hydroxylapatite lattice.

**Figure 3:**
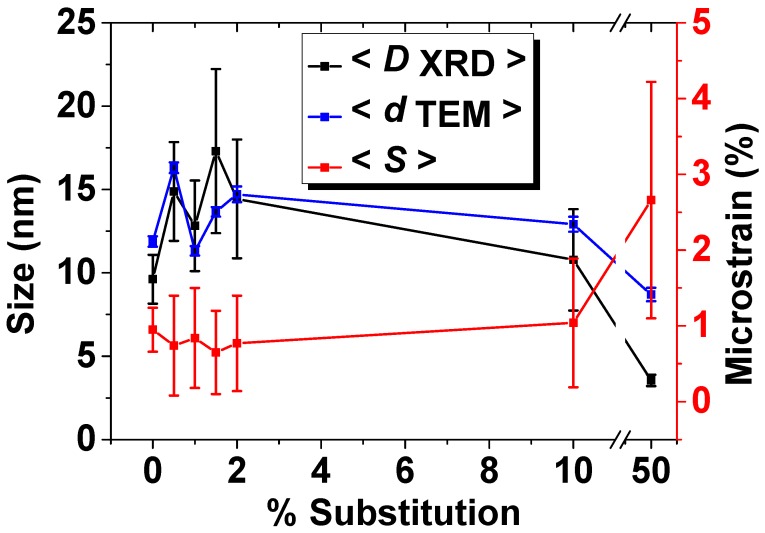
The estimated crystallite size, lattice microstrain and degree of crystallinity.

The estimation of crystallite size, lattice microstrain and degree of crystallinity are shown in Table 2 and Figure 3.

Up to 1.5%, the introduction of Eu^3+^ in HAp lattice induces an increase of crystallite size from 9.62 nm to 17.3 nm and a decrease of lattice microstrain from 0.95% to 0.65%. For a substitution degree of more than 1.5%, there may be observed a decrease of the crystallite size to 3.55 nm and an increase of lattice microstrain to 2.66% in the case of maximum substitution degree. In what concerns the crystallinity degree (Table 2), as it may be appreciated qualitatively from the profile of the peaks, the crystallinity decreases when the substitution degree increases.

### 3.2. SEM Analysis

**Figure 4:**
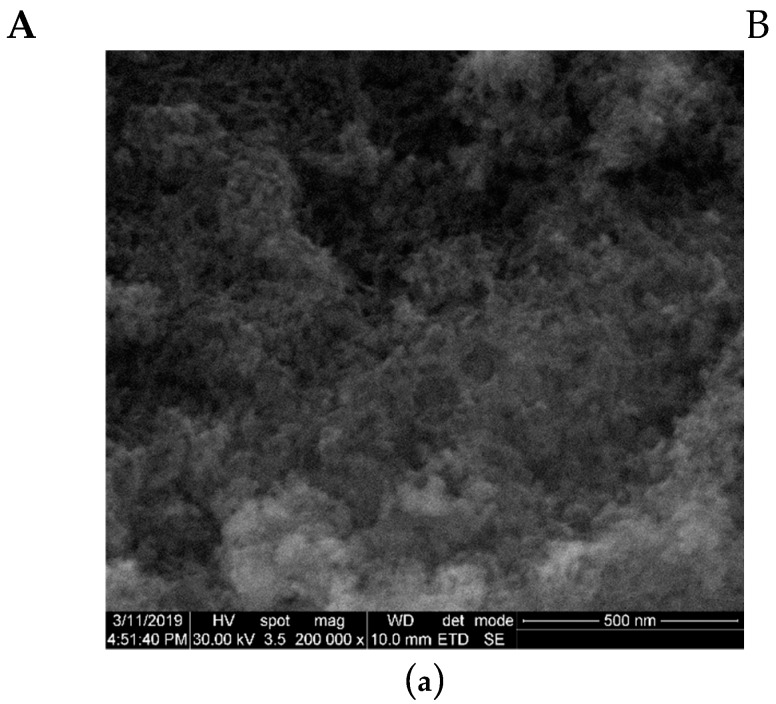

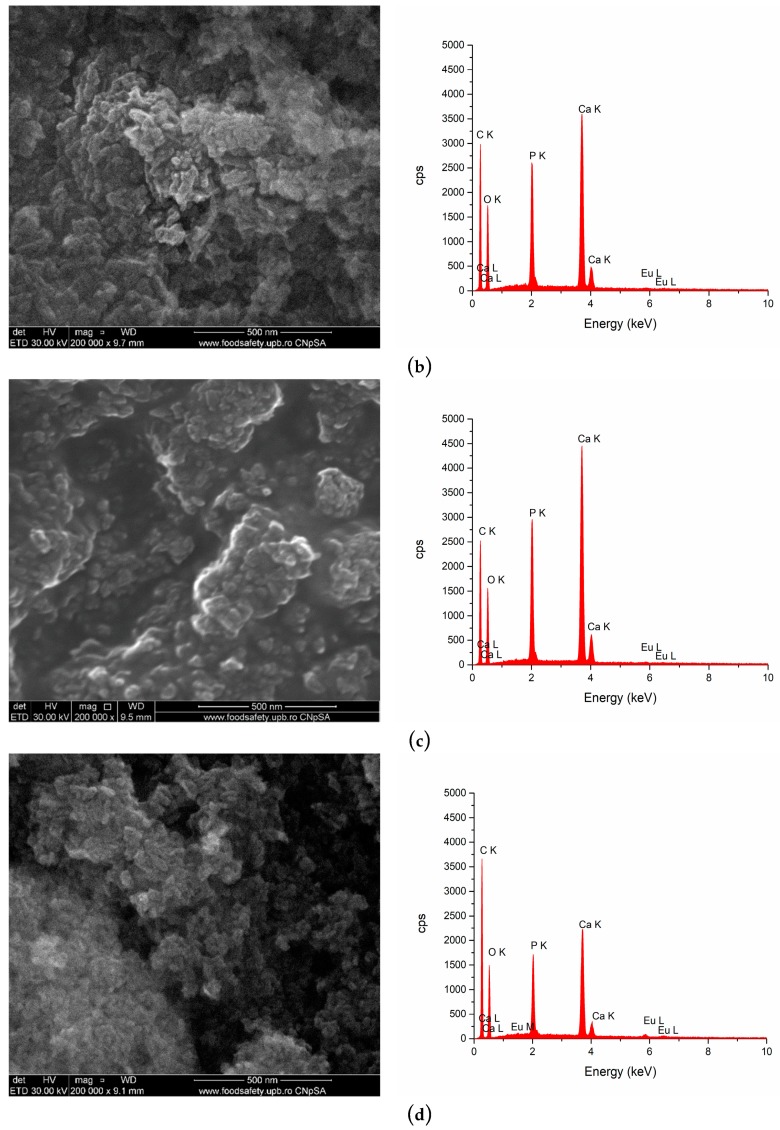

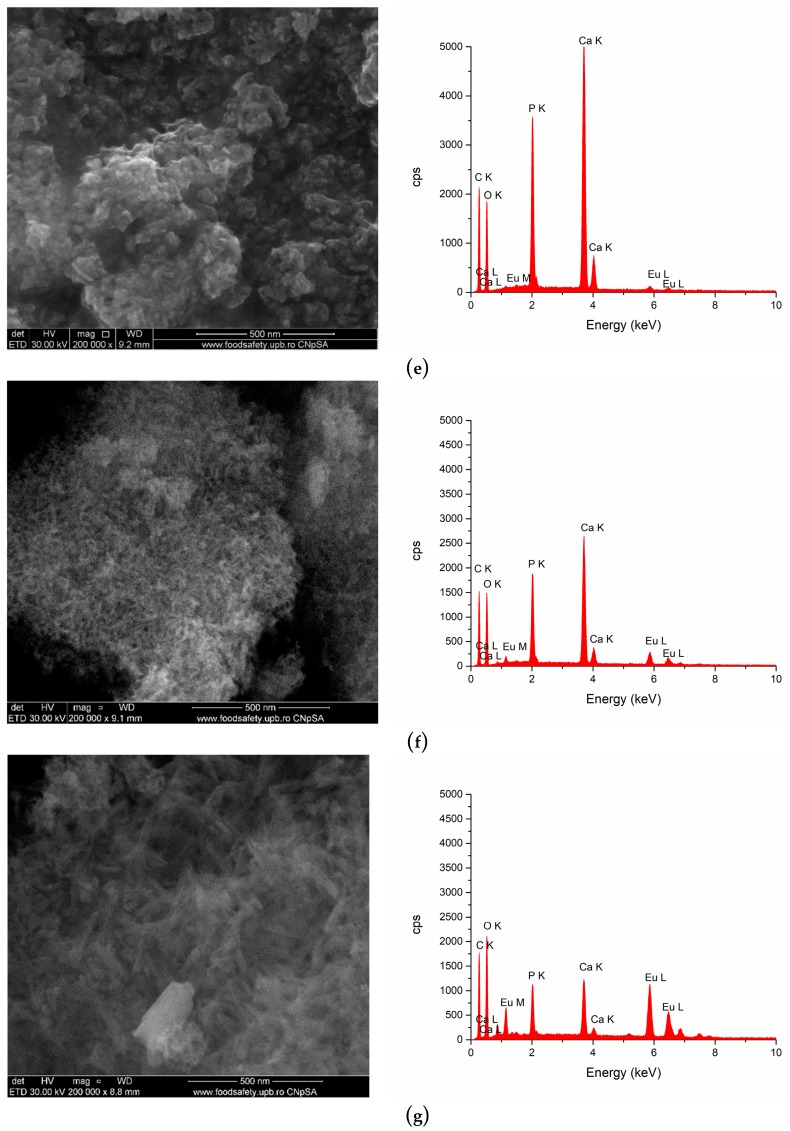
The SEM images (column **A**) and EDX spectra (column **B**) of pure HAp and Eu_X_HAp samples (**a**) HAp, (**b**) Eu0.5HAp, (**c**) Eu1HAp, (**d**) Eu1.5HAp, (**e**) Eu2HAp, (**f**) Eu10HAp, (**g**) Eu50HAp.

Figure 4 shows the SEM images (column A) and EDX spectra (column B) of pure HAp (Figure 4a) and Eu_X_HAp samples (Figure 4b).

It can be seen that the doping with Eu^3+^ has little influence on the morphology of substituted HAp compared to the pure HAp. SEM images (Figure 4, column A) reveal quasi-spherical (at low dopant concentration) and acicular (mostly after 10% Eu) particles with width in the range of 6–16 nm. Due to high surface area, agglomerates are present in all samples. The EDX spectra of studied samples (Figure 4, column B) confirm the presence of all elements specific to Eu-doped HAp powders: calcium (Ca), phosphor (P), oxygen (O) and europium (Eu).

### 3.3. TEM Analysis

**Figure 5:**
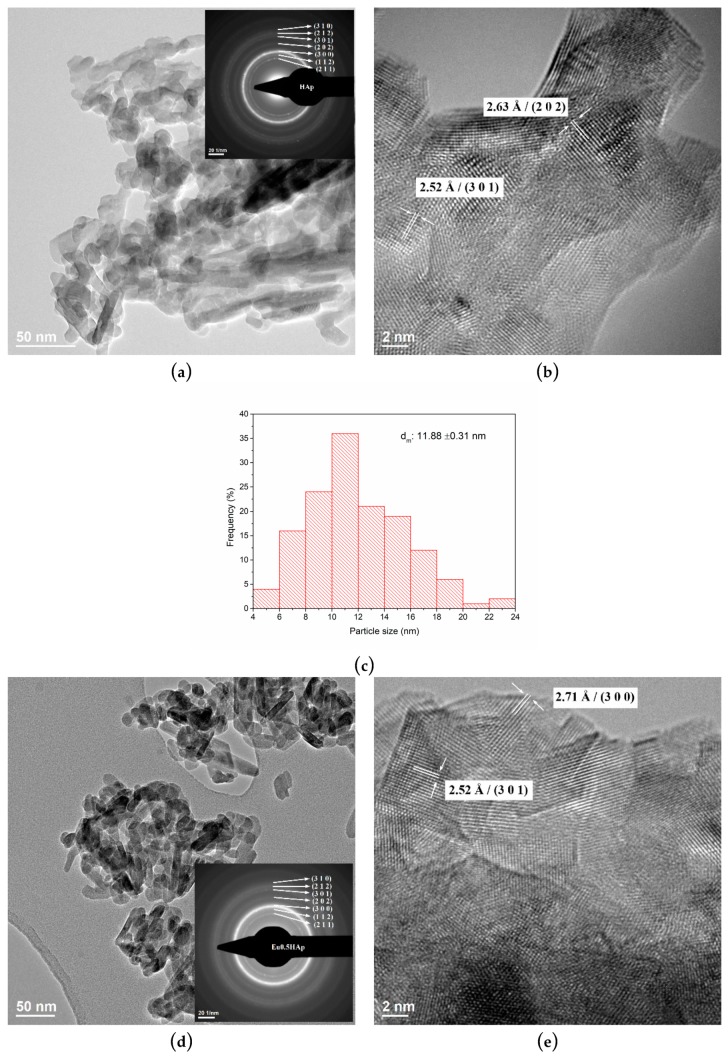

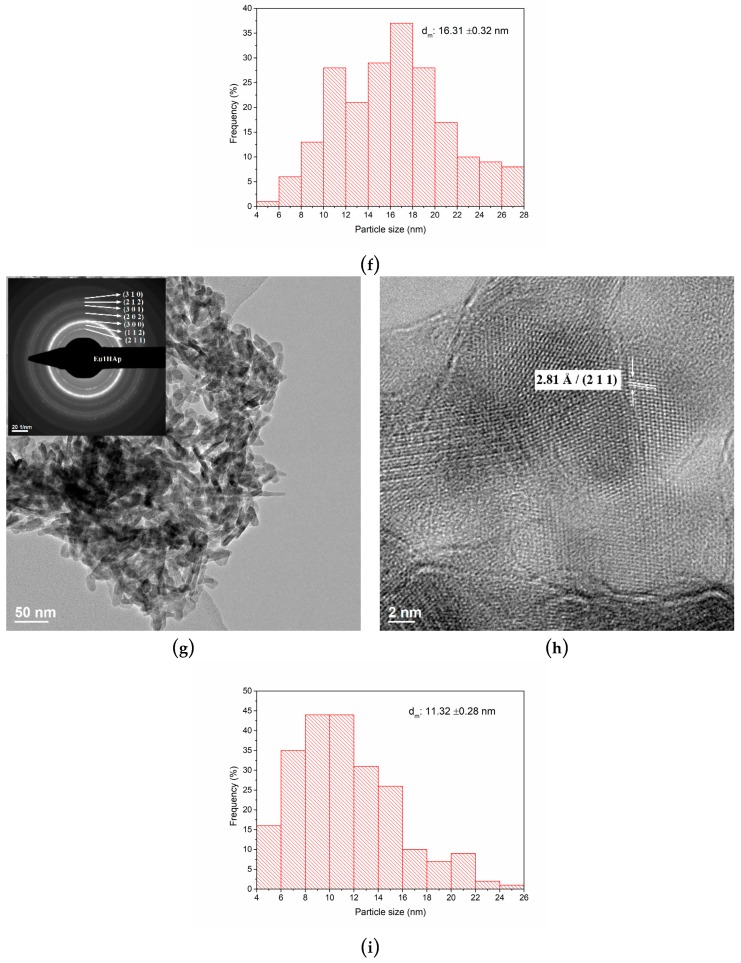

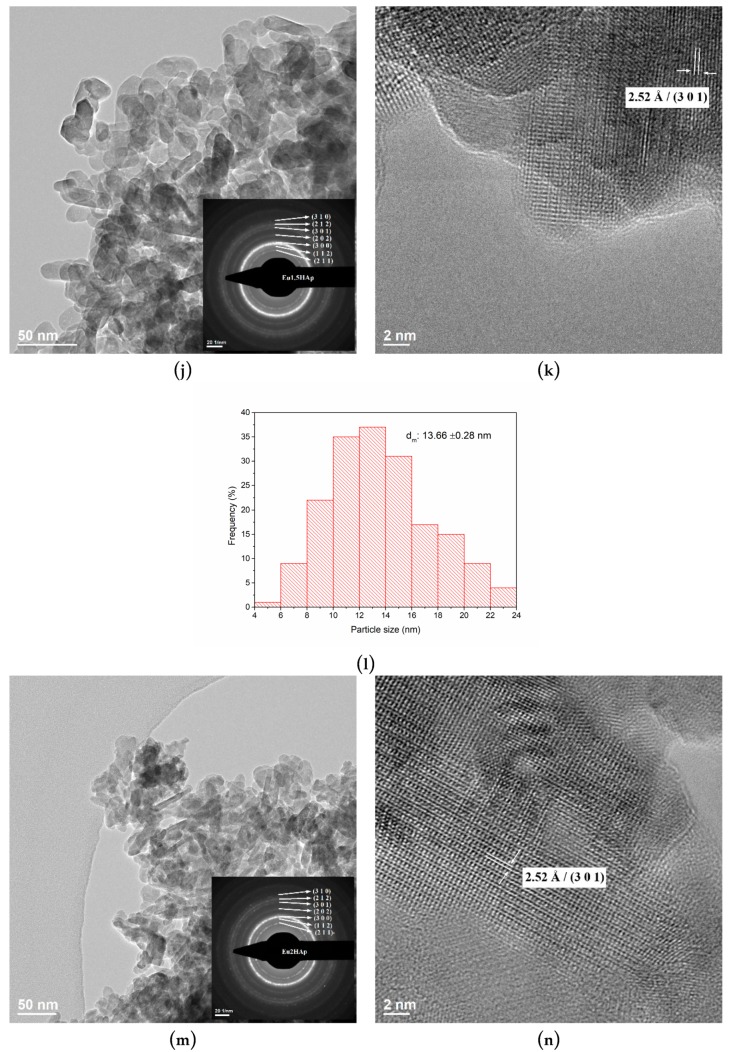

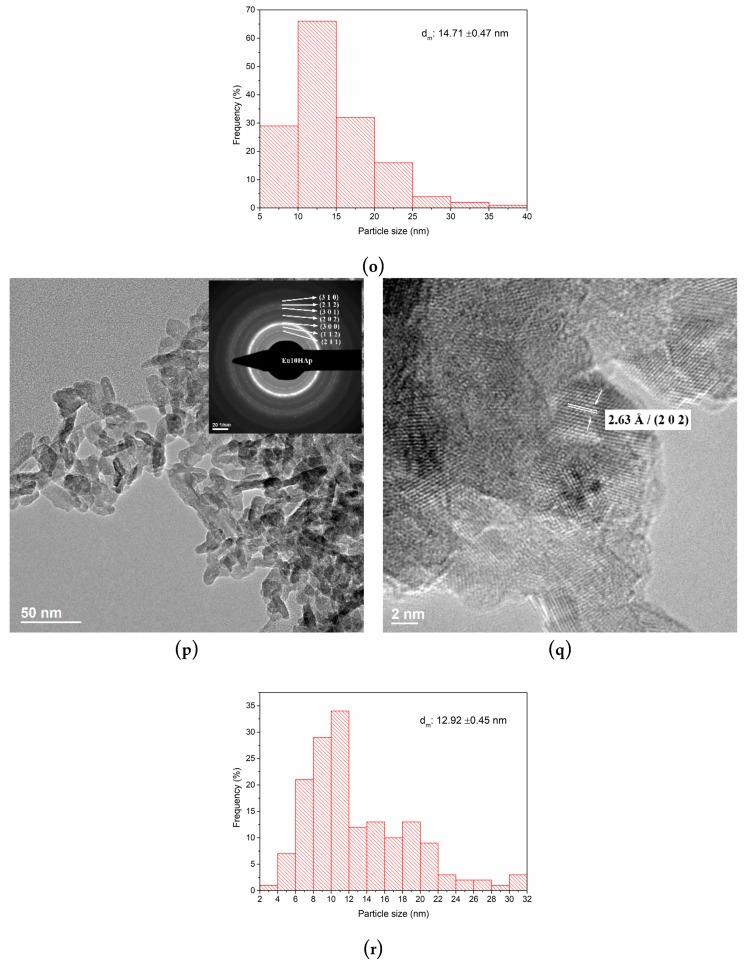

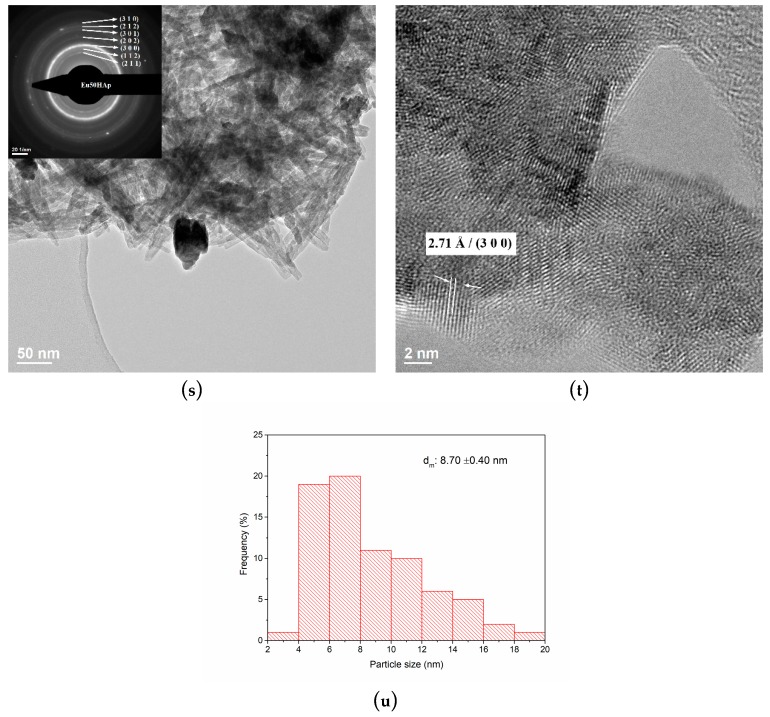
The TEM and HRTEM images, SAED patterns and particle size distribution of pure HAp and Eu_X_HAp samples (**a**–**c**) HAp, (**d**–**f**) Eu0.5HAp, (**g**–**i**) Eu1HAp, (**j**–**l**) Eu1.5HAp, (**m**–**o**) Eu2HAp, (**p**–**r**) Eu10HAp, (**s**–**u**) Eu50HAp.

Figure 5 shows the bright field TEM, HRTEM images, SAED patterns and particle size distribution of pure HAp and Eu_X_HAp at different Eu^3+^ concentrations.

From the bright field TEM images presented in Figure 5a,d,g,j,m,p,s it can be observed that the doping level does not affect the the sample morphologies from 0 to 10% Eu^3+^ doping, the only visible modification if for the 50% Eu^3+^ doping and it is related to the width (smaller) and length (longer) of the acicular particles. The SEM micrographs together with TEM images confirmed the tendency to form agglomerate due to probably the nanometric dimensions of particles. The size distribution presented in Figure 5c,f,i,l,o,r,u is depended on the dopant concentration. The HRTEM images of the Eu-doped HAp powders are also presented in Figure 5 which allows us to see that the particles are well crystalize and the measured distance of the Miller indices correspond to the hexagonal hydroxylapatite.

### 3.4. FTIR Spectra

**Figure 6:**
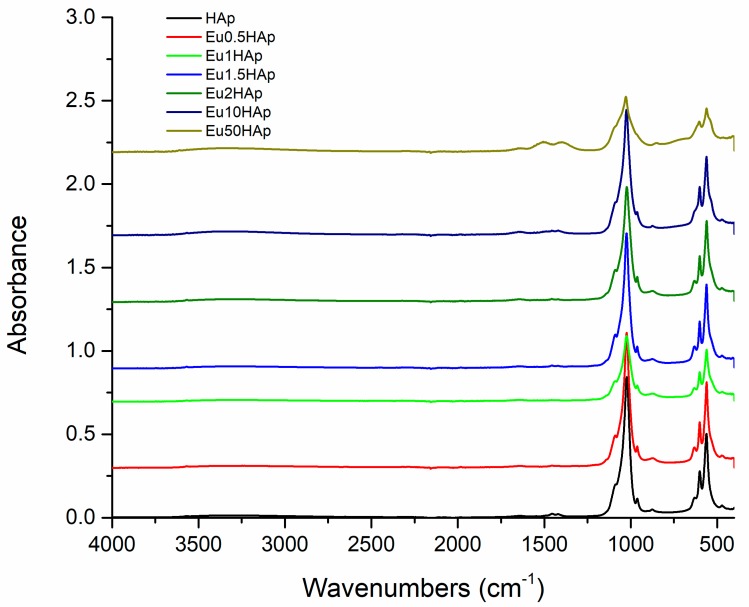
FTIR spectra of Eu_X_HAp samples.

The FTIR spectra of Eu_X_HAp samples with various europium concentrations are shown in Figure 6.

The broad band in the region 3200–3400 cm^−1^ corresponds to [OH]^-^ bands of adsorbed water. The strong band at 632 cm^-1^ (presented in all FTIR spectra) corresponds to [OH]^-^ arising from stretching vibrational mode [50,51]. The typical bands attributed to [PO_4_]^3−^ can be also found. The bands at around 1090 cm^−1^ and about 1040 cm^−1^ may be assigned to the antisymetric stretching ν_3_[PO_4_]^3−^ of P-O bond, while the band at 962 can be due to the symmetric stretching ν_1_[PO_4_]^3−^. The 602 cm^−1^ and 564 cm^−1^ bands appear from ν_4_[PO_4_]^3−^ of P-O bond. The band at 475 cm^−1^ can be attributed to the ν_2_[PO_4_]^3−^ [52]. In all spectra of Eu_X_HAp a band at 875 cm^−1^ was detected, and is due to [HPO_4_]^2−^ ions [53]. The intensity of the phosphate bands decreases with increase of europium concentration. The bands at 475 and 962 cm^−1^ progressively reduced their intensities with the increase of europium concentration, and disappear in Eu50HAp sample, as already suggested also by XRD results. The different possible mechanisms of Ca substitution with Eu are not fully comprehended and need further studies

### 3.5. Raman Spectroscopy

**Figure 7:**
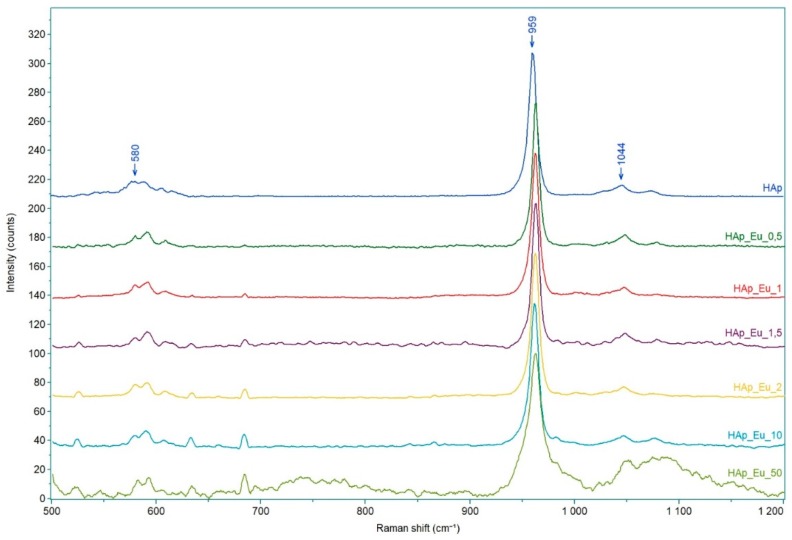
Raman spectra of Eu_X_HAp samples.

The Raman spectra of Eu_X_HAp samples are shown in Figure 7.

As it can be seen from Figure 7 all analyzed powders present bands attributed to PO_4_^3−^ group [54]. Raman spectrum of pure HAp shows a very intense band at 959 cm^−1^ attributed to symmetric stretching mode of PO_4_^3−^, which is the characteristic peak of HAp. The asymmetric stretching and symmetric bending modes of PO_4_^3−^ are also observed at 580 cm^−1^ and 1044 cm^−1^ [53]. Raman spectra of Eu_X_HAp powders show the internal modes of the frequency ν_1_ PO_4_^3−^ tetrahedral, which appears at 962 cm^−1^ and it corresponds to the symmetric stretching of P–O bonds [55]. There are observed several other bands at 580 cm^−1^ and 610 cm^−1^ attributed to ν_4_ PO_4_^3−^, and 1048 cm^−1^ and 1080 cm^−1^, respectively, attributed to ν_3_ PO_4_^3−^. The intensity of the characteristic peak of HAp, compared to the other secondary peaks, decreases with increasing of the europium content.

### 3.6. UV-Vis and PL Spectra

**Figure 8:**
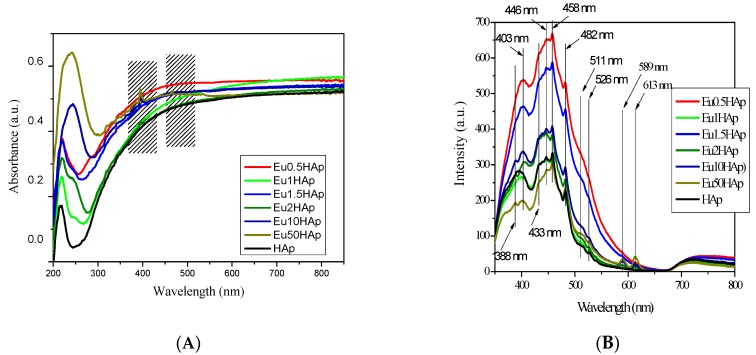
(**A**) UV-Vis absorption spectra of Eu_X_HAp at different Eu-concentrations (**B**) Room-temperature photoluminescence spectra of Eu_X_HAp at different Eu-concentrations.

The electronic spectra of Eu_X_HAp samples (Figure 8) contain several bands, which increase in their intensity with increasing of europium content.

The pure HAp has an absorption peak in UV at 218 nm. The 242 nm (41322 cm^−1^) absorption peak is absent in HAp, but is increasing in intensity from a weak shoulder in Eu0.5HAp to a strong, broad peak in Eu50HAp. This is a charge-transfer band characteristic to Eu^3+^ in oxides [56]. The band which appears at 395 nm, close to the visible light domain, corresponds to the ^7^F_0_ → ^5^L_6_ transition, and is the most intense transition of europium (III) in UV-Vis absorption spectra [57]. Another three bands appear at 465, 526 and 535 nm, assignable to ^7^F_0_ → ^5^D_2_; ^7^F_0_ → ^5^D_1_; and ^7^F_1_ → ^5^D_1_, transitions respectively [56]. The last peak, at 535 nm, is belonging to a group called “hot” bands because it can be observed only at room temperatures or higher since it requires the thermal population of ^7^F_1_ level (at room temperature ~35% of ions are populating this level, rest being on ^7^F_0_ ground state) [56].

Figure 8B presents the photoluminescence emission spectra of Eu_X_HAp samples excited with 320 nm wavelength. Usually, HAp sample shows three strong emission peaks at 458 nm with two shoulders (433 nm and 446 nm), 403 nm and 482 nm, respectively, associated with various oxygen defects. The intensity of luminescence for HAp is enhanced by the presence of small quantities of Eu^3+^ ions. The effect is stronger for small numbers of dopant ions and is decreasing as the quantity of Eu^3+^ ions is increasing.

Over the fluorescence maxima of HAp, the emission spectra of europium are superposed. The peak at 389 nm is due to the ^7^F_0_ → ^5^L_6_ electronic transition, the peak at 512 nm is due to the ^7^F_0_ → ^5^D_1_ electronic transition and the peak at 526 nm is due to the ^7^F_1_ → ^5^D_1_ electronic transition. The characteristic emission peaks at 589 nm and 613 nm are due to the ^5^D_0_ → ^7^F_1_ and ^5^D_0_ → ^7^F_2_ electronic transition, indicating a red fluorescence [58]. For higher Eu^3+^ concentration even the 648 nm emission peak of ^5^D_0_→ ^7^F_3_ becomes visible. As expected, when the europium doping concentration increased, the characteristic emission intensity at 589 and 613 nm are also increased.

Beside the two dominant peaks, the weak ^5^D_0_ → ^7^F_0_ transition from 579 nm (which can be observed in emission spectra of Eu50HAp) is related with Eu^3+^ ions distributed on Ca^2+^ sites of the apatitic structure [59]. The dominant emission peaks present no shift due to modification of Eu^3+^ concentration, the most intense being the hypersensitive ^5^D_0_ → ^7^F_2_ transition from 613 nm. This feature shows the potential application of the Eu_X_HAp compounds to be tracked or monitored by the characteristic luminescence.

## 4. Biocompatibility and Cytotoxicity of Eu_X_HAp Photoluminescent Ceramic Materials

**Figure 9:**
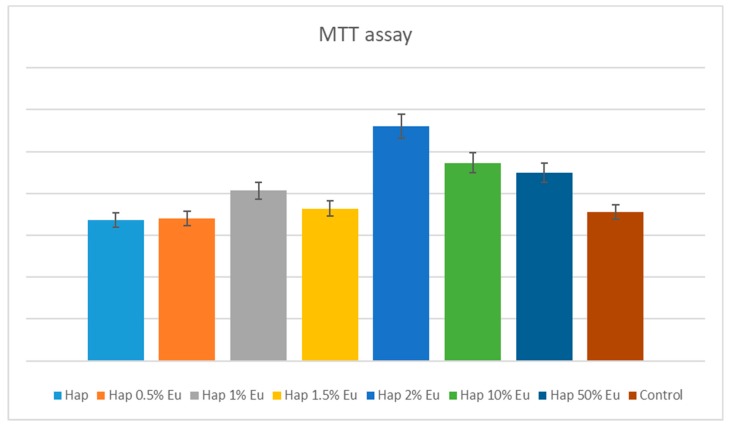
MTT assay showing the viability of AFSC in the presence of the Eu_X_HAp ceramic materials: HAp, Eu05HAp, Eu1HAp, Eu1.5HAp, Eu2HAp, Eu10HAp, Eu50HAp, and control (cell only).

Cytotoxic effect of Eu_X_HAp was evaluated by measuring the metabolic activity of AFSC using MTT assay. Eu_X_HAp biomaterials did not have cytotoxic effect, the absorption values being near or higher compared to the control sample. Furthermore, the Eu_X_HAp increases cellular metabolism, suggesting that it stimulates cell proliferation. The proliferation increases between 12%–58%, depending on the sample, highest increase being observed for Eu2HAp (58%) (Figure 9). The results are similar to other research groups that showed the viability of HGF-1 fibroblast was decreased in the Eu10Hap after 48 h. Naderi et al. 2012 [60] tested different concentrations of nanohydroxyapatite from 2 to 0.002  mg/mL on gingiva-derived fibroblast cell line (HGF-2) at 24, 48, and 72 h, and concluded that after 24 h high doses of nanohydroxyapatite have cytotoxic effect on gingival-derived fibroblasts suggesting that cytotoxicity is dependent on the cell line [60,61].

**Figure 10:**
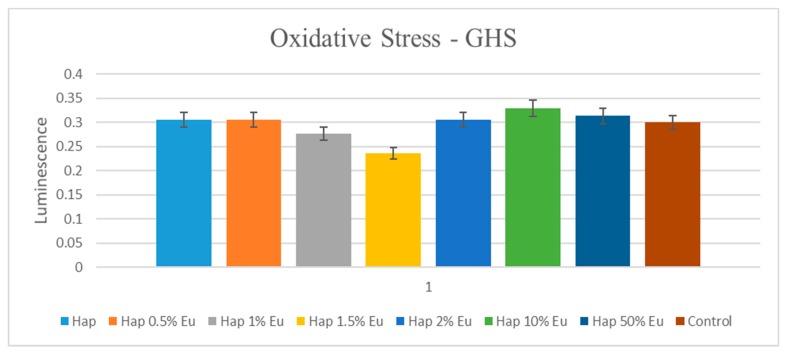
GSH assay showing the oxidative stress of AFSC in the presence of the Eu_X_HAp ceramic materials: HAp, Eu05HAp, Eu1HAp, Eu1.5HAp, Eu2HAp, Eu10HAp, Eu50HAp and control (cell only).

Glutathione, an oxidative stress marker, is capable of preventing cellular damage caused by reactive oxygen species, such as free radicals, peroxides, lipid peroxides and heavy metals. In the presence of Eu_X_HAp biomaterials, AFSC responded similarly to control cells, indicating that the analysed materials did not induce cellular stress (Figure 10). Furthermore, the morphology of AFSC was investigated by fluorescence microscopy using CMTPX cell tracker for long-term tracing of living cells. Cellular metabolism is active, as shown in microscopy images, cells absorbing CMTPX fluorophore in the cytoplasm, suggesting that they are viable.

**Figure 11:**
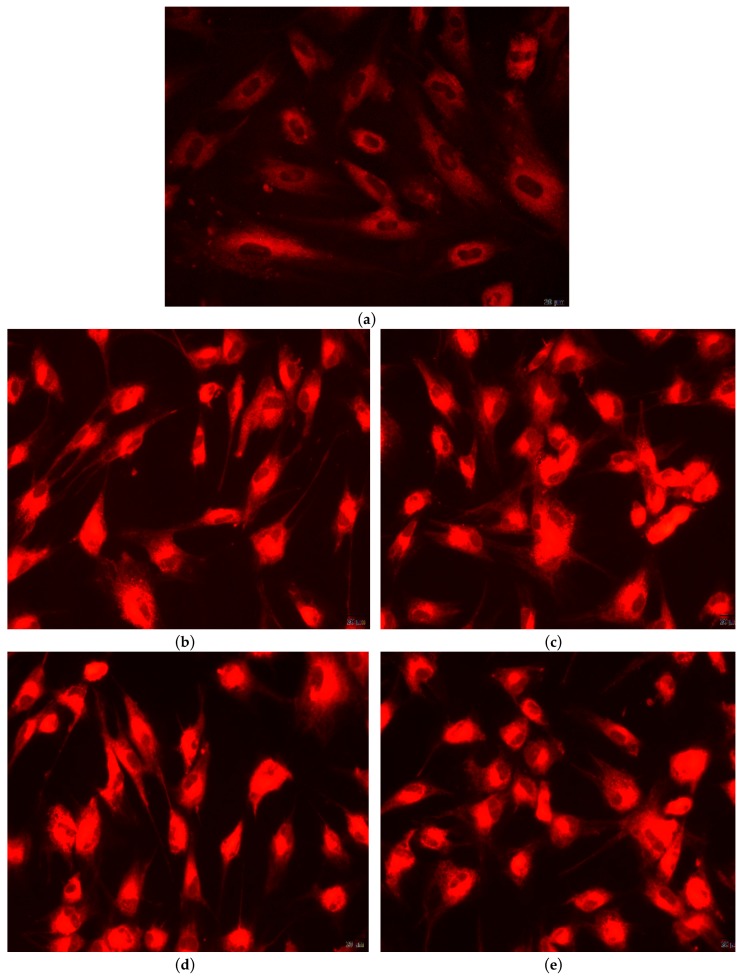

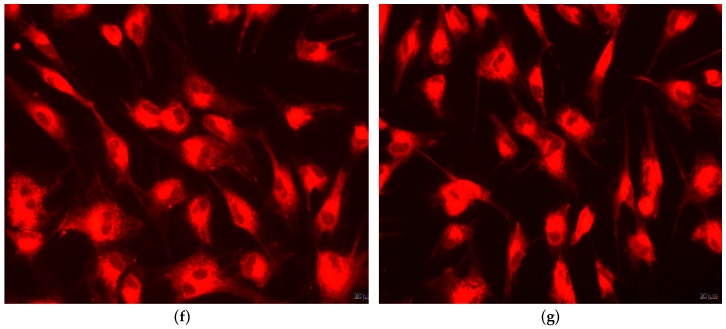
Fluorescence images of Eu_X_HAp samples coloured with CMTPX fluorophore (**a**) Control sample, (**b**) Eu0.5HAp, (**c**) Eu1HAp, (**d**) Eu1.5HAp, (**e**) Eu2HAp, (**f**) Eu10HAp, (**g**) Eu50HAp.

After 5 days in the presence of Eu_X_HAp, AFSCs presents a normal morphology with fibroblastic-like characteristic appearance (Figure 11). Fluorescent images show that AFSC cells are viable, no dead cells or cell fragments are observed, more the cells spread filopodia to move and establish contacts with neighboring cells, suggesting that AFSC have an active phenotype.

## 5. Conclusions

In recent years, a special attention has been created regarding to multimodal imaging. This technique represents various imaging modalities in the manner of photoluminescence and magnetic resonance imaging that are generally connected in an individual diagnostic step. If we talk about multimodal contrast agents that can be used or marker for biomedical imaging with luminescence, literature data provides information on the use of doped hydroxyapatite with rare earth elements like europium of nanometric dimensions.

The aim of the present study was to obtain europium-doped nano hydroxylapatite by using a simple method of synthesis (co-precipitation method) and, thereafter characterize by physico-chemical techniques and also by biological point of view because of the fact that the obtained material has medical application.

The results after the XRD analysis shows that the obtained material is represented by pure hexagonal phase of hydroxylapatite, when using up to 10% dopant Eu^3+^ ions concentration, but there is a correlation between HAp and Eu^3+^ by the interference of Eu^3+^ ions with HAp crystal structure. About the morphology of HAp doped with Eu^3+^ it can be concluded that there is a limited modification after the addition of Eu^3+^ and also by the EDS spectra is confirmed the presence of Eu^3+^ in all obtained materials. Typically, the HAp sample shows three strong emission peaks, but the photoluminescence intensities is enhanced by the characteristic Eu^3+^ peaks with the presence of Eu^3+^ ions. This feature is required and necessary to have a good action in medical imaging.

From the biological point of view, it can be seen by the MTT assay results that the obtained material supports the proliferation process of the amniotic fluid stem cells. Also, the best results about the viability characteristic is offered by the Eu2HAp material which can be a standard for the doped HAp, because after the addition of more Eu^3+^ ions there is a small evidence about the cellular viability that can decrease. As a complete evaluation, the qualitative information by the fluorescence microscopy gives characteristic about the biocompatibility of the material in light of the fact that after 5 days of incubation with amniotic fluid stem cells, no dead cells are observed.

Therefore, there is a fine line about the using doped HAp with Eu^3+^ as fluorescent or multimodal contrast agent, but the results can be a promising start due to its characteristics but there is a need for the further investigation.

## Figures and Tables

**Table 1:** Calculated crystallite size (*D*) values and degree of crystallinity (X_c_) of pure HAp and europium doped hydroxylapatite with various amount of Eu.

No.	Samples	D/nm	S/%	X_c_/%
1	HAp	9.62 ± 1.46	0.95 ± 0.29	33.93
2	Eu0.5HAp	14.88 ± 2.96	0.74 ± 0.66	26.25
3	Eu1HAp	12.82 ± 2.72	0.84 ± 0.66	24.11
4	Eu1.5HAp	17.3 ± 4.92	0.65 ± 0.55	22.97
5	Eu2HAp	14.44 ± 3.56	0.77 ± 0.63	22.63
6	Eu10HAp	10.78 ± 3.04	1.04 ± 0.85	23.03
7	Eu50HAp	3.55 ± 0.34	2.66 ± 1.56	21.87

**Table 2:** Unit cell parameters *a*, *c*, *V* and agreement indices for hydroxylapatite HAp and Eu-doped HAp (with concentration of Eu^3+^ of 0.5, 1, 1.5, 2, 10 and 50%).

Sample	a [Å]	c [Å]	V [Å^3^]	*R_exp_*	*R_p_*	*R_wp_*	*χ^2^*
HAp	9.434979 ± 0.001401	6.8803397 ± 0.001042	530.6588	3.4323	3.99694	5.19277	2.28891
Eu0.5HAp	9.440372 ± 0.002067	6.878142 ± 0.001529	530.8601	3.96601	4.82349	6.554	2.7309
Eu1HAp	9.445147 ± 0.002444	6.879144 ± 0.001839	531.4745	3.92894	4.95602	6.61378	2.83367
Eu1.5HAp	9.439496 ± 0.001874	6.879717 ± 0.001413	530.883	3.7995	4.38638	5.76083	2.29889
Eu2HAp	9.440372 ± 0.002067	6.878142 ± 0.001529	530.8601	3.96601	4.82349	6.554	2.7309
Eu10HAp	9.443276 ± 0.002227	6.879882 ± 0.001665	531.321	3.88457	4.43662	5.82612	2.24943
Eu50HAp	9.544463 ± 0.021918	6.315845 ± 0.023992	498.2704	3.02385	3.11688	4.08783	1.82753
